# Mortality After Non–ST-Segment Elevation Myocardial Infarction: Impact of Left Ventricular Function and Coronary Artery Disease

**DOI:** 10.1016/j.jacadv.2025.102155

**Published:** 2025-09-16

**Authors:** Nina Stødkilde-Jørgensen, Kevin KW. Olesen, Christine Gyldenkerne, Malene K. Hansen, Bjarne L. Nørgaard, Troels Thim, Roni R. Nielsen, Michael Maeng

**Affiliations:** a Department of Cardiology, Aarhus University Hospital, Aarhus, Denmark; b Department of Clinical Medicine, Aarhus University, Aarhus, Denmark

**Keywords:** coronary artery disease, ejection fraction, NSTEMI, prognosis

## Abstract

**Background:**

Non–ST-segment elevation myocardial infarction (NSTEMI) due to coronary artery disease (CAD) can lead to reduced left ventricular ejection fraction (LVEF) and increased mortality. However, the interplay between LVEF and obstructive CAD on mortality has not been examined in patients with NSTEMI.

**Objectives:**

The purpose of this study was to examine the combined prognostic impact of LVEF and CAD on mortality following an NSTEMI.

**Methods:**

We included patients referred to coronary angiography due to first-time NSTEMI with obstructive CAD registered in the Western Denmark Heart Registry. Patients were grouped according to LVEF (>50%, 41% to 50%, and ≤40%) and the extent of obstructive CAD defined by vessel disease (VD) (1VD, 2VD, and 3VD). Five-year cumulative incidence proportions and HRs of mortality were calculated. Excess mortality was assessed by comparison with a sex- and age-matched general population cohort.

**Results:**

In total, 8,770 patients with NSTEMI and obstructive CAD were included between 2010 and 2021. The lowest 5-year mortality was observed for patients with LVEF >50% and 1VD (9%), followed by an increase with decreasing LVEF and increasing CAD. The highest mortality was observed for patients with LVEF ≤40% and 3VD (46%; adjusted HR 3.05; 95% CI: 2.51-3.70). This relationship was confirmed by comparison with the matched general population, where patients with LVEF ≤40% and 3VD had 24% higher absolute mortality.

**Conclusions:**

In patients with NSTEMI, the combined information on LVEF and the extent of obstructive CAD was associated with an increasing 5-year mortality. A comparison with a matched general population confirmed the findings.

Most patients with non–ST-segment elevation myocardial infarction (NSTEMI) undergo coronary angiography (CAG), where the extent of coronary artery disease (CAD) is assessed.^1^ By definition, patients with NSTEMI have myocardial damage related to infarction, which can lead to reduced left ventricular ejection fraction (LVEF) and heart failure. Heart failure is often caused by CAD, and it is well-known that a reduced LVEF and increasing extent of CAD separately are associated with a poor prognosis after NSTEMI.^2^^,^^3^

Substantial progress has been made in treating heart failure by introducing modern medical therapies.^4^, ^5^, ^6^ Similarly, the treatment of myocardial infarction and CAD has improved by faster and more effective interventions, coupled with an increased emphasis on postevent risk factor modifications and promotion of a more cardioprotective lifestyle, potentially reducing the risk of an adverse clinical outcome.^1^^,^^7^, ^8^, ^9^, ^10^

However, among patients with a first-time NSTEMI, the contemporary risk of all-cause mortality associated with LVEF combined with the extent of obstructive CAD remains unclear. Insights into this area may guide clinicians on risk stratification. This study aimed to: 1) assess the combined prognostic impact of LVEF and the extent of obstructive CAD on all-cause mortality following a first-time NSTEMI in a contemporary cohort; and 2) compare mortality with a matched general population cohort.

## Methods

### Study design and data sources

This prospective cohort study was based on patients registered in the Western Denmark Heart Registry (WDHR), a database containing detailed information, such as LVEF, CAD, smoking, and body mass index, on all patients examined with CAG in Western Denmark since 1999.^11^ The data obtained from the WDHR were cross-linked with data on redeemed prescriptions acquired from the Danish National Prescription Registry, data on diagnoses and admissions from the Danish National Patient Registry, data on relevant blood tests from the Register of Laboratory Results for Research, and data on time of death from the Danish Civil Registration System.^11^ Every Danish citizen has a unique 10-digit identification number, allowing cross-linking of these registries.

### Participants

All included patients were ≥18 years of age and underwent first-time CAG between 2010 and 2021, as registered in WDHR. In Denmark, the clinical standard is to perform a CAG within 48 to 72 hours for stable patients with NSTEMI, while unstable patients are examined acutely. Patients were excluded if the procedure was registered as elective or if the referral diagnosis was not NSTEMI. Furthermore, patients were excluded if they had a previous diagnosis of myocardial infarction, percutaneous coronary intervention (PCI), or coronary artery bypass grafting (CABG), missing data on LVEF or CAD, or LVEF <10% or >75%. Finally, patients with diffuse or no obstructive CAD were excluded, leaving patients with first-time NSTEMI and obstructive CAD for inclusion.

### Variables

The main outcome was all-cause mortality (hereafter referred to as mortality), while an outcome of cardiovascular mortality as defined in the SCORE2 model^12^ was analyzed in a supplemental analysis. Patients were placed into groups according to their LVEF and extent of obstructive CAD. The included LVEF (hereafter referred to as >50%, 41% to 50%, or ≤40%) was measured during the admission leading up to the index CAG. The middle group of LVEF was defined as 41% to 50% instead of the standard range of 41% to 49% to ensure enough patients in all 3 groups. At the beginning of the inclusion period, obstructive CAD was defined as >50% stenosis, which was later supplemented by measuring the fractional flow reserve and instantaneous wave-free ratio. The extent of obstructive CAD was expressed as the number of affected coronary vessels (vessel disease [VD]; 1VD, 2VD, or 3VD). The WDHR follows the conventional reporting of obstructive CAD, where vessel involvement is defined anatomically by the 3 main coronary arteries (left anterior descending artery, left circumflex artery, and right coronary artery) and where significant left main disease is classified as 2VD. Diffuse CAD without focal stenosis was considered nonobstructive. For descriptive analyses, information on prescriptions redeemed from 180 to 7 days before the index CAG was included. Detection of comorbidities up to 30 days after the index CAG (except heart failure, which was registered up until the index CAG) was based on either the International Classification of Diseases-10th revision codes using a full look-back in the Danish National Patient Registry or a recording in the WDHR (Supplemental Table 1). A diagnosis of diabetes could also be based on a redeemed prescription for antidiabetic medication (not including sodium-glucose transport protein 2 inhibitors) within 180 days before to 30 days after the index CAG.

### General population cohort comparison

Patients were compared with a matched general population cohort. The patients were matched individually on sex and age in a 1:5 ratio to randomly sampled general population cohort controls selected from the Danish Civil Registration System, with the possibility of the same individual being sampled more than once.^13^ The controls were without a previous diagnosis of myocardial infarction, PCI, or CABG registered in the Danish National Patient Registry, but 1.4% (N = 646) had a diagnosis of heart failure.

### Statistical methods

Follow-up of outcomes began 30 days after the index CAG to exclude procedure-related outcomes. Patients were followed until death, emigration, the end of follow-up (December 31, 2022), or a maximum of 5 years. Baseline characteristics are shown as medians with first and third quartiles (Q_1_ and Q_3_), mean values with SDs, or frequencies with percentages. Analyses by LVEF, CAD, LVEF + CAD, and 30-day revascularization are presented. Five-year cumulative incidence proportions were estimated with the Kaplan-Meier estimator for mortality and the Aalen-Johansen estimator for cardiovascular mortality, accounting for the competing risk of noncardiovascular mortality. Proportional hazards assumptions were assessed visually using log-log plots and were found not to be violated. HRs were calculated using cause-specific Cox regression analyses and presented as unadjusted or adjusted for the following categorical variables: sex, age (<65 years, 65-74 years, or >74 years), hypertension, previous ischemic stroke, peripheral artery disease, atrial fibrillation, CAD (1VD, 2VD, or 3VD), smoking (never/former or active), and diabetes. Missing data were assumed to be missing at random and handled using multiple imputations. A cubic spline regression analysis with 3 knots was performed using the median LVEF of 55% as a reference and depicted 5-year mortality related to LVEF as a continuous variable. Finally, we tested for interaction between LVEF as a continuous variable and the extent of CAD. *P* < 0.05 was considered significant.

Additional analyses include an analysis grouped by sex or age (18-64 years or ≥65 years), a sensitivity analysis excluding all patients diagnosed with heart failure more than 30 days before the index CAG, an analysis grouped by 30-day revascularization, and a sensitivity analysis expanding the multivariable regression model to also include revascularization within 30 days after the index CAG. Estimates are presented with 95% CIs. The analyses were conducted using Stata/MP v. 18.0 (StataCorp LP).

## Ethical approval

The study was approved by a regional branch of the Danish Data Protection Agency (record no. 1-16-02-193-18). Upon approval by the regional data protection authorities (record no. 14-45-70-24-22), patient consent requirements were waived.

## Results

**Figure 1:**
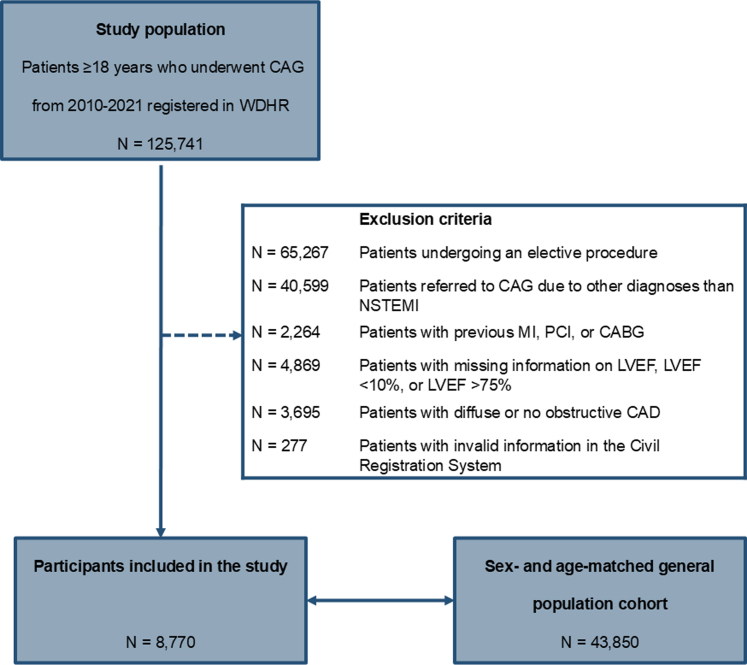
**Participants** Figure 1 illustrates the steps leading to the inclusion of 8,770 patients and 43,850 matched controls. CABG = coronary artery bypass grafting; CAG = coronary angiography; CAD = coronary artery disease; LVEF = left ventricular ejection fraction; MI = myocardial infarction; NSTEMI = non–ST-segment elevation myocardial infarction; PCI = percutaneous intervention; WDHR = Western Denmark Heart Registry.

Between 2010 and 2021, 125,741 patients underwent a first-time CAG in Western Denmark. Among these, 8,770 patients with first-time NSTEMI and obstructive CAD were eligible for this study, and 43,850 sex- and age-matched controls from the general population were included for comparison (Figure 1). Within the 5-year follow-up, 1,403 patients and 5,083 controls died. Apart from emigration (N = 31), no patients were lost to follow-up.

### Baseline characteristics

Table 1 presents the baseline characteristics by LVEF >50% (N = 5,063; 58%), LVEF 41% to 50% (N = 2,163; 25%), and LVEF ≤40% (N = 1,544; 18%). Two-thirds of the patients were male, and the median age at first-time NSTEMI increased from 65 years (Q_1_-Q_3_: 56-74 years) to 73 years (Q_1_-Q_3_: 65-80 years) with decreasing LVEF. In patients with LVEF >50%, 1VD was more prevalent (59%), while the number of obstructed vessels was more evenly distributed in patients with LVEF ≤40%, being 34%, 30%, and 36% for 1VD, 2VD, and 3VD, respectively. Between 0 and 30 days after the CAG, 6,647 (76%) were treated with PCI, while 1,073 (12%) were treated with CABG. Cardiac risk factors were predominant in all 3 groups of LVEF, especially family history of premature ischemic heart disease (24% to 37%), smoking (28% to 30%), hypertension (57% to 66%), and diabetes (16% to 31%). In the group of patients with LVEF ≤40%, 30% had a diagnosis of heart failure before the NSTEMI, while the corresponding prevalence was 1% in those with LVEF >50%. The median time from diagnosis of heart failure to the index CAG was 4 days (Q_1_-Q_3_: 2-43 days).

**Table 1:** Baseline Characteristics of NSTEMI Patients by LVEF

Characteristic	LVEF >50% (n = 5,063)	LVEF 41%-50% (n = 2,163)	LVEF ≤40% (n = 1,544)
Age in y, median (Q_1_-Q_3_)	65 (56-74)	69 (59-78)	73 (65-80)
Male, n (%)	3,636 (71.8)	1,546 (71.5)	1,018 (65.9)
BMI (kg/m^2^), median (Q_1_-Q_3_)	27 (25-30)	27 (24-30)	26 (23-29)
Family history of premature IHD, n (%)	1,864 (36.8)	719 (33.2)	371 (24.0)
Smoking, n (%)	1,516 (29.9)	611 (28.2)	458 (29.7)
Aortic systolic BP (mm Hg), median (Q_1_-Q_3_)	140 (122-160)	135 (120-155)	125 (110-144)
Aortic diastolic BP (mm Hg), median (Q_1_-Q_3_)	70 (60-80)	70 (60-80)	65 (58-75)
Heart rate, beats/min (Q_1_-Q_3_)	73 (65-82)	76 (67-88)	83 (72-95)
Comorbidities, n (%)			
Hypertension	2,865 (56.6)	1,347 (62.3)	1,011 (65.5)
Heart failure before index CAG	59 (1.2)	135 (6.2)	468 (30.3)
Previous ischemic stroke	128 (2.5)	92 (4.3)	105 (6.8)
Previous hemorrhagic stroke	14 (0.3)	11 (0.5)	11 (0.7)
Peripheral artery disease	275 (5.4)	197 (9.1)	220 (14.2)
Atrial fibrillation/flutter	496 (9.8)	322 (14.9)	300 (19.4)
Diabetes	822 (16.2)	462 (21.4)	482 (31.2)
COPD	409 (8.1)	251 (11.6)	206 (13.3)
Coronary artery disease, n (%)			
1VD	3,006 (59.4)	1,053 (48.7)	522 (33.8)
2VD	1,246 (24.6)	604 (27.9)	464 (30.1)
3VD	811 (16.0)	506 (23.4)	558 (36.1)
Medication, n (%)			
Statin	1,092 (21.6)	492 (22.7)	504 (32.6)
Beta-blocker	617 (12.2)	340 (15.7)	310 (20.1)
ACE inhibitor	730 (14.4)	360 (16.6)	315 (20.4)
ARB	1,008 (19.9)	476 (22.0)	347 (22.5)
MRA	58 (1.1)	45 (2.1)	49 (3.2)
SGLT2 inhibitor	32 (0.6)	19 (0.9)	23 (1.5)
Furosemide	240 (4.7)	195 (9.0)	199 (12.9)
Calcium-blocker	949 (18.7)	449 (20.8)	381 (24.7)
Thiazide	498 (9.8)	198 (9.2)	190 (12.3)
Aspirin	636 (12.6)	346 (16.0)	374 (24.2)
P2Y12 inhibitor	158 (3.1)	111 (5.1)	74 (4.8)
VKA	96 (1.9)	75 (3.5)	67 (4.3)
DOAC	100 (2.0)	70 (3.2)	52 (3.4)
Insulin	173 (3.4)	121 (5.6)	140 (9.1)
Noninsulin	413 (8.2)	244 (11.3)	243 (15.7)
Blood test values			
eGFR, mean (SD)	80.9 (19.8)	74.8 (22.3)	67.6 (23.9)
Cholesterol, total (mmol/L), mean (SD)	5.2 (1.2)	5.1 (1.2)	4.7 (1.3)
LDL-cholesterol (mmol/L), mean (SD)	3.2 (1.1)	3.0 (1.1)	2.8 (1.1)
HDL-cholesterol (mmol/L), mean (SD)	1.3 (0.4)	1.3 (0.4)	1.3 (0.4)
Triglyceride (mmol/L), mean (SD)	2.0 (1.4)	1.9 (1.3)	1.6 (1.0)
HbA1c, median (Q_1_-Q_3_)	39 (36-43)	40 (36-44)	41 (38-49)
Revascularization day 0-30 after CAG, n (%)			
Days to revascularization, median (Q_1_-Q_3_)	0 (0-0)	0 (0-1)	0 (0-3)
PCI	3,961 (78.2)	1,618 (74.8)	1,068 (69.2)
CABG	571 (11.3)	278 (12.9)	224 (14.5)

Baseline characteristics for the 3 groups of LVEF.

Comorbidities are registered until 30 days after the index CAG, except heart failure, which is only included until the day of the CAG.

ACE inhibitor = angiotensin-converting-enzyme inhibitor; ARB = angiotensin receptor blocker; BMI = body mass index; BP = blood pressure; CABG = coronary artery bypass grafting; CAG = coronary angiography; COPD = chronic obstructive pulmonary disease; DOAC = direct oral anticoagulant; eGFR = estimated glomerular filtration rate; HDL = high-density lipoprotein; IHD = ischemic heart disease; LDL = low-density lipoprotein; LVEF = left ventricular ejection fraction; MRA = mineralocorticoid receptor antagonist; NSTEMI = non–ST-segment elevation myocardial infarction; PCI = percutaneous coronary intervention; Q_1_-Q_3_ = first and third quartiles; SGLT2 inhibitor = sodium-glucose transport protein 2 inhibitor; VD = vessel disease; VKA = vitamin K antagonist.

In Supplemental Table 2, baseline characteristics are presented by 1VD (N = 4,581; 52%), 2VD (N = 2,314; 26%), and 3VD (N = 1,875; 21%). Increasing age (65 years vs 72 years), percentage of male patients (69% vs 74%), and comorbidities were observed, with the extent of CAD increasing from 1VD to 3VD. Between 0 and 30 days after the CAG, revascularization by PCI was most common in patients with 1VD (85%) and least common in those with 3VD (49%), while the opposite was found for CABG rates (Supplemental Table 2). Similar results were found when grouping by both LVEF and the extent of obstructive CAD (Supplemental Table 3).

Patients who underwent revascularization within 30 days were younger (67 years vs 71 years), were more often male (73% vs 57%), and had fewer comorbidities than those who did not undergo revascularization (Supplemental Table 4). In the 180 days following the index CAG, usage of especially statins, aspirin, P2Y12 inhibitors, and beta-blockers was high in all groups, both when grouped by LVEF and when grouped by the extent of CAD (Supplemental Tables 5 and 6).

### Mortality related to LVEF level and the extent of obstructive CAD in patients with NSTEMI

**Figure 2:**
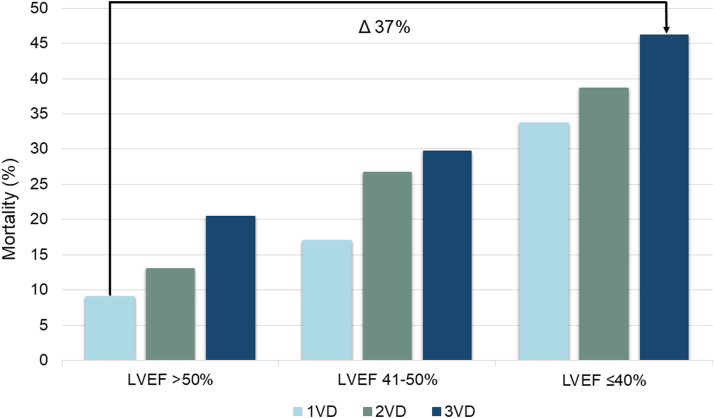
**Cumulative 5-Year Mortality by LVEF and Extent of Obstructive CAD** Figure 2 depicts the 5-year mortality grouped by LVEF, and obstructive CAD expressed as 1-3VD. An increase in risk is observed with decreasing LVEF and increasing extent of CAD, adding up to a risk difference of 37.1% between LVEF >50% and 1VD, and LVEF 10% to 40% and 3VD. VD = vessel disease; other abbreviations as in Figure 1.

**Figure 3:**
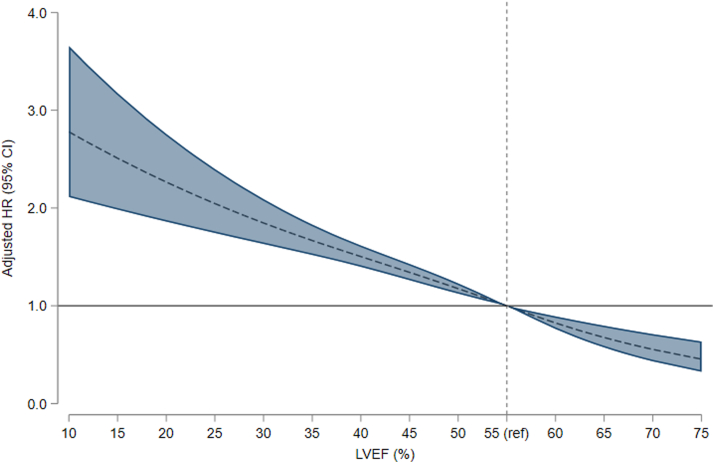
**Spline Regression Analysis of LVEF and Mortality Risk** Figure 3 depicts a spline regression analysis of the 5-year mortality risk by reported LVEF. The median LVEF of 55% is set as a reference, and the darker areas represent 95% CI. The HR was adjusted for sex, age, hypertension, previous ischemic stroke, peripheral artery disease, CAD, atrial fibrillation, smoking, and diabetes. Abbreviations as in Figure 1.

**Figure 4:**
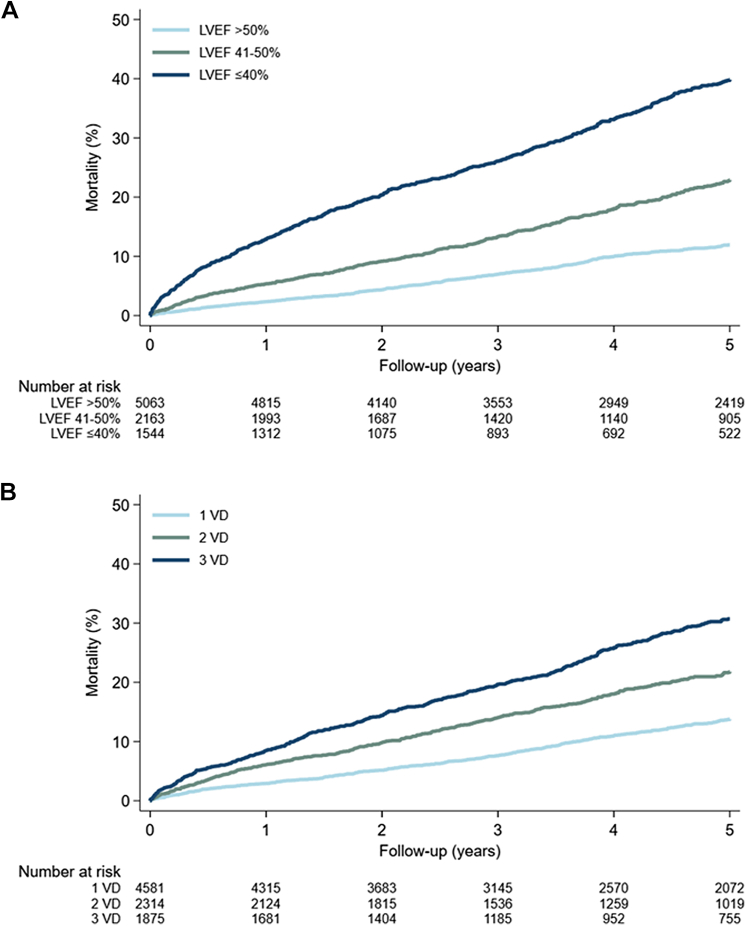
Mortality During Follow-Up Grouped by LVEF and Obstructive CAD Figure 4 depicts the 5-year cumulated mortality risk and risk tables: (A) grouped by LVEF, with the risk reaching almost 40% for patients with the lowest LVEF, and (B) grouped by the extent of CAD expressed as 1VD, 2VD, and 3VD, with the risk reaching more than 30% for 3VD. Abbreviations as in Figures 1 and 2.

Combining LVEF and the extent of obstructive CAD demonstrated an increase in 5-year mortality from 9% for LVEF >50% and 1VD to 46% for LVEF ≤40% and 3VD (adjusted HR: 3.05; 95% CI: 2.51-3.70, Table 2). The increase in 5-year mortality corresponds to a risk difference of 37% from LVEF >50% and 1VD to LVEF ≤40% and 3VD (Central Illustration, Figure 2). We observed no interaction between LVEF and CAD extent (*P*_interaction_ = 0.10). A sensitivity analysis excluding those with a heart failure diagnosis dated more than 30 days before the index CAG showed consistent results (Supplemental Table 7). When grouped by revascularization within 30 days, a pattern was evident of higher adjusted HRs among patients who did not undergo revascularization, with the exception of those with LVEF ≤40% and 1VD (Supplemental Tables 8 and 9). Adjusting for revascularization yielded similar results as the main findings (Supplemental Table 10). A spline regression examining LVEF separately from CAD showed a steep increase in the adjusted HR of mortality, with a decreasing LVEF (Figure 3). By the 3 groups of LVEF, 5-year mortality increased from 12% for LVEF >50% to 40% for LVEF ≤40% (adjusted HR: 2.32; 95% CI: 2.04-2.65) (Supplemental Table 11, Figure 4A) while the corresponding 5-year cardiovascular mortality increased from 3% to 17% (adjusted HR: 3.23; 95% CI: 2.59-4.03) (Supplemental Table 11). An additional analysis was performed to evaluate the impact of sex and age on mortality. In the group with the lowest LVEF, the analysis demonstrated a 5-year mortality of 20% (adjusted HR: 3.61; 95% CI: 2.48-5.26) for those aged 18 to 64 years and 46% (adjusted HR: 2.36; 95% CI: 2.06-2.71) for those older than 64 years. Men and women shared largely similar risks in all 3 groups of LVEF (Supplemental Table 12).

**Table 2:** 5-Year Mortality Among Patients With First-Time NSTEMI Grouped by LVEF and Extent of Obstructive CAD

LVEF and CAD Group	Patients	Deaths	5-Year CIP	Unadjusted HR	Adjusted HR Using LVEF >50% and 1VD as Ref^a^	Adjusted HR Using 1VD as Ref^a^
LVEF >50%						
1VD	3,006	214	9.1% (8.0-10.4)	Ref	Ref	Ref
2VD	1,246	133	13.0% (11.0-15.2)	1.50 (1.21-1.86)	1.15 (0.92-1.43)	1.15 (0.92-1.43)
3VD	811	136	20.5% (17.5-23.8)	2.43 (1.96-3.01)	1.50 (1.20-1.87)	1.50 (1.20-1.87)
LVEF 41%-50%						
1VD	1,053	141	17.0% (14.5-19.8)	1.98 (1.60-2.45)	1.56 (1.26-1.94)	Ref
2VD	604	127	26.7% (22.8-31.1)	3.11 (2.50-3.87)	1.91 (1.53-2.38)	1.18 (0.92-1.50)
3VD	506	129	29.7% (25.6-34.4)	3.87 (3.11-4.82)	2.03 (1.63-2.54)	1.25 (0.98-1.59)
LVEF ≤40%						
1VD	522	141	33.7% (29.2-38.7)	4.26 (3.44-5.26)	2.43 (1.96-3.02)	Ref
2VD	464	160	38.7% (34.1-43.8)	5.82 (4.74-7.14)	3.17 (2.57-3.90)	1.29 (1.03-1.63)
3VD	558	222	46.2% (41.7-51.0)	6.79 (5.62-8.19)	3.05 (2.51-3.70)	1.30 (1.05-1.61)

CAD = coronary artery disease; CIP = cumulative incidence proportion; other abbreviations as in Table 1.

Patients and deaths are listed as counts, CIP as percentages with 95% CI, and HR as ratios with 95% CI.

Mortality grouped by LVEF and the extent of obstructive CAD.

Adjusted HRs are shown both with LVEF >50% and 1VD as a reference and with 1VD as a reference within each group of LVEF. An increasing 5-year CIP was observed with a decreasing LVEF and increasing extent of CAD.

a Adjusted for sex, age, hypertension, previous ischemic stroke, peripheral artery disease, atrial fibrillation, smoking, and diabetes.

**Central Illustration:**
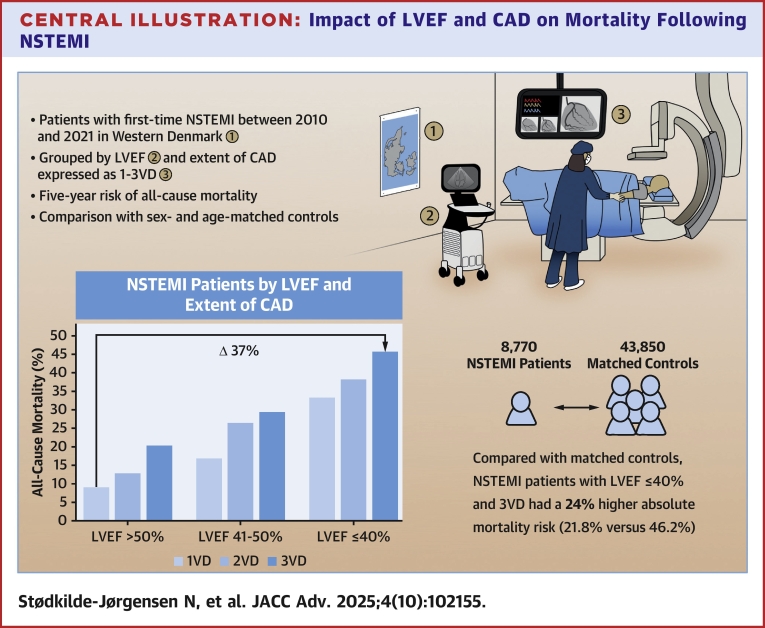
**Impact of LVEF and CAD on Mortality Following NSTEMI** Abbreviations as in Figures 1 and 2.

Examination of CAD revealed an association between the presence of multivessel obstructive CAD and 5-year mortality: The risk increased from 14% for 1VD to 31% for 3VD (adjusted HR: 1.35; 95% CI: 1.19-1.54) for mortality (Supplemental Table 11, Figure 4B), and from 4% for 1VD to 13% for 3VD (adjusted HR: 2.08; 95% CI: 1.66-2.61) for cardiovascular mortality (Supplemental Table 11).

### Comparison to a sex- and age-matched general population cohort

To surpass the age differences between LVEF groups and evaluate excess mortality, sex- and age-matched general population cohort controls were included for comparison. Grouped by the patients’ LVEF and extent of obstructive CAD, an association was confirmed between an increasing 5-year mortality and a decreasing LVEF and increasing extent of obstructive CAD for both patients and controls. However, the increase in mortality was much steeper for the patients with NSTEMI, evidenced by a similar risk of −0.4% (95% CI: −1.7 to 0.8) for LVEF >50% and 1VD, which increased to a risk difference of 24% (95% CI: 19.6-29.2) for LVEF ≤40% and 3VD (Table 3).

**Table 3:** 5-Year Mortality Among Patients Grouped by LVEF and Extent of Obstructive CAD Compared With Controls

LVEF and CAD Group	Patients	Deaths	5-Year CIP	Controls	Deaths	5-Year CIP	Risk Difference
LVEF >50%							
1VD	3,006	214	9.1% (8.0-10.4)	15,030	1,179	9.6% (9.0-10.1)	−0.4% (−1.7 to 0.8)
2VD	1,246	133	13.0% (11.0-15.2)	6,230	612	11.5% (10.6-12.4)	1.5% (−0.7 to 3.8)
3VD	811	136	20.5% (17.5-23.8)	4,055	525	15.3% (14.2-16.6)	5.2% (1.7-8.5)
LVEF 41%-50%							
1VD	1,053	141	17.0% (14.5-19.8)	5,265	590	13.7% (12.7-14.8)	3.3% (0.6-6.0)
2VD	604	127	26.7% (22.8-31.1)	3,020	418	16.8% (15.4-18.4)	9.9% (5.7-14.1)
3VD	506	129	29.7% (25.6-34.4)	2,530	419	19.3% (17.8-21.1)	10.4% (5.7-15.1)
LVEF ≤40%							
1VD	522	141	33.7% (29.2-38.7)	2,610	406	18.5% (16.9-20.3)	15.2% (10.2-20.1)
2VD	464	160	38.7% (34.1-43.8)	2,320	410	20.5% (18.8-22.4)	18.2% (13.1-23.3)
3VD	558	222	46.2% (41.7-51.0)	2,790	524	21.8% (20.2-23.5)	24.4% (19.6-29.2)

Patients/controls and deaths are listed as counts and CIP as percentages with 95% CI.

This Table presents 5-year mortality grouped by LVEF, and the extent of obstructive CAD expressed as 1VD, 2VD, and 3VD in patients compared with their sex- and age-matched general population controls. With decreasing LVEF and increasing extent of CAD among patients, an increasing risk difference between the 2 groups was observed.

Abbreviations as in Tables 1 and 2.

## Discussion

Patients with NSTEMI represent a large group of patients in day-to-day clinical practice. These patients undergo echocardiography to assess LVEF and CAG to evaluate CAD. While left ventricular dysfunction and CAD are both associated with mortality, the interplay between LVEF and the extent of obstructive CAD vs 5-year mortality has not yet been examined in patients with first-time NSTEMI. In the present study, we evaluated the impact of LVEF as measured before CAG and obstructive CAD on mortality in a large, contemporary NSTEMI cohort. Within each LVEF group, the increasing extent of obstructive CAD was strongly associated with mortality, and a combined assessment of LVEF and obstructive CAD led to an increase in 5-year all-cause and cardiovascular mortality. These findings remained evident even when excluding patients with an older heart failure diagnosis. As patients with a normal LVEF were younger than those with a lower LVEF, the observed associations could be due to unmeasured confounding. However, our comparison with a sex- and age-matched general population comparison cohort showed an increasing excess mortality when LVEF decreased, and the extent of obstructive CAD increased. Taken together, LVEF and the extent of obstructive CAD are 2 parameters that, when combined, are strongly associated with all-cause mortality and excess mortality.

### The impact of LVEF and the extent of obstructive CAD on mortality

Efficacious therapies for heart failure with reduced LVEF have been implemented following multiple pivotal randomized trials over the past 30 years, for example, beta-blockers, angiotensin-converting enzyme inhibitors, mineralocorticoid receptor antagonists, sodium-glucose transport protein 2 inhibitors, and cardiac resynchronization therapy.^6^ Simultaneously, the introduction of statins, aspirin, P2Y12 inhibitors, and coronary revascularization represents crucial landmarks in treating CAD and myocardial infarction.^1^^,^^14^^,^^15^ These developments have improved survival in patients with heart failure or CAD.^16^^,^^17^ Nonetheless, both conditions continue to impose serious consequences. This is evidenced by our finding of 5-year mortality spanning from 9% to 46%, despite a high rate of revascularization being performed during the first 30 days and high treatment adherence in the first 6 months following CAG.

When looking beyond NSTEMI patients, previous studies examining the impact of reduced LVEF on mortality have shown consistent results.^2^^,^^18^^,^^19^ Thus, a large cohort study examining trends in more than 190,000 patients with heart failure reported a 5-year mortality of approximately 45% at the end of their study period in 2017.^18^ Similarly, a meta-analysis of 60 studies, including 1.5 million patients with heart failure, described 5-year survival rates reaching approximately 60% in 2000-2009.^19^ The impact of the extent of CAD on mortality is well-documented. For example, in a cohort study of 55,000 patients with myocardial infarction, the authors reported increasing incidence rates of recurrent nonfatal myocardial infarction, nonfatal stroke, or cardiovascular death, with increasing extent of CAD, as well as a similar prevalence of multi-VD compared to our study (41% vs 48%).^20^ However, the novelty in our study relies on the combined assessment of LVEF and CAD, which led to a more refined gradual risk of mortality, and the use of the sex- and age-matched general population, which removed at least some of the unmeasured residual confounding associated with the age differences observed in the NSTEMI cohort.

### THE Combined impact of LVEF and the extent of obstructive CAD on mortality

Due to their intertwined nature, we found that exploring the complete spectrum of LVEF and obstructive CAD was appropriate when assessing prognosis. Nonetheless, we could not identify similar publications as the available data were few, and the conclusions were inconsistent: In an analysis of 2,707 patients with advanced chronic systolic heart failure included in the BEST (Beta-blocker Evaluation of Survival Trial), a history of CAD (ie, not based on CAG) was associated with increased mortality.^21^ An analysis of 591 patients with heart failure and either preserved or reduced ejection fraction suggested that a history of CAD was only associated with prognostic impact in patients with reduced LVEF.^22^ A publication from the Swedish Heart Failure Registry reported that a history of CAD was associated with increased mortality across all heart failure types. However, the study divided patients into a dichotomous ischemic or nonischemic cardiomyopathy and was limited to a heart failure cohort.^23^ Two cohorts with 376 and 4,128 patients with heart failure and preserved LVEF both showed that the presence of angiographically documented CAD was associated with increased mortality.^24^^,^^25^ Finally, 3 large studies^3^^,^^26^^,^^27^ have demonstrated that the presence of CAD, as verified by CAG, was strongly associated with mortality in patients with a reduced LVEF.

Thus, the combined impact of LVEF as measured before CAG and the extent of obstructive CAD on mortality had not been examined in patients with first-time NSTEMI, and our large cohort study provides novel insights into this area.

### Strengths and limitations

A key strength of this study is the contribution to available knowledge by examining the interplay between different levels of LVEF and the extent of obstructive CAD. Furthermore, Danish health registries allow the examination of long-term individual-level data with virtually no loss to follow-up. Crosslinking different registries provides a substantial amount of data for each patient, making analyses more robust and allowing for subgroup analyses. Moreover, universal health care offered in Denmark limits the risk of selection bias. We acknowledge that there are potential limitations. First, the comparison with a matched general population cohort does not consider the possibility of subclinical or undiagnosed cardiovascular disease in the comparison cohort, which could lead to an underestimation of the true risk differences and bias the results toward the null hypothesis. Second, although LVEF is a standard measurement in cardiology, it represents a single point in time and is subject to interobserver variability, as well as potential improvement after revascularization and guideline-directed treatment. Furthermore, we acknowledge that the categorization of LVEF, though reflecting a generally accepted clinical categorization, is a simplification of a continuous parameter as illustrated in Figure 3. Third, in patients without a prior echocardiography, it is impossible to distinguish between a hitherto unknown reduced LVEF present before the NSTEMI and a reduced LVEF caused by the NSTEMI. Fourth, we grouped CAD according to the number of coronary arteries with obstructive CAD. This simplifies the spectrum of CAD, and the results must be interpreted in this context. Fifth, handling of nonculprit lesions may differ between hospitals, regions, and countries. In Western Denmark, it has been the strategy to aim for complete revascularization since the FRISC (FRagmin and Fast Revascularization during InStability in Coronary artery disease) II study.^28^ This includes careful consideration of PCI of chronic total occlusions. Finally, some parameters related to the NSTEMI were unavailable to us, such as troponin levels, the use of vasoactive agents or mechanical support, and the presence of cardiogenic shock, which could have affected the findings.

## Conclusions

In this Danish all-comers cohort of patients with first-time NSTEMI, decreased LVEF and increased extent of obstructive CAD were associated with increased 5-year mortality. The combined information from the LVEF assessment and the extent of obstructive CAD was associated with an increase in 5-year mortality, and a comparison with a matched general comparison cohort confirmed this relationship. Our study thus provides valuable clinical knowledge for more precise risk stratification of patients with NSTEMI.

Perspectives **COMPETENCY IN MEDICAL KNOWLEDGE:** Disease severity and risk assessment in NSTEMI is a continuum, making individualized treatment plans after CAG challenging. LVEF and the extent of CAD, which are parameters already measured, are key determinants of clinical outcomes in patients with a first-time NSTEMI.

**TRANSLATIONAL OUTLOOK:** Integrating LVEF and the extent of CAD into standard risk models based on, for example, baseline risk variables, comorbidity burden, and electrocardiogram changes may improve accuracy in risk assessment. This could provide more objective, patient-specific tools for future risk assessment and patient management after NSTEMI.

## Funding support and author disclosures

This work was funded by a grant from the 10.13039/501100009708Novo Nordisk Foundation, Denmar (grant number NNF22OC0074083). Dr Olesen has received a grant from the Danish Cardiovascular Academy (grant no. CPD5Y-2022001-HF), which is funded by the 10.13039/100007405Danish Heart Association and the 10.13039/501100009708Novo Nordisk Foundation. Dr Nørgaard has received unrestricted research grants from the 10.13039/501100009708Novo Nordisk Foundation. Dr Thim has received lecture fees from Terumo and Chiesi. Dr Maeng is supported by a grant from the 10.13039/501100009708Novo Nordisk Foundation (grant number NNF22OC0074083); has received lecture and/or advisory board fees from AstraZeneca, Bayer, Boehringer Ingelheim, Bristol Myers Squibb, and Novo Nordisk; has received research grants from Philips, Bayer, and Novo Nordisk; has received a travel grant from Novo Nordisk; has ongoing institutional research contracts with Janssen, Novo Nordisk, and Philips; and has equity interests in Novo Nordisk, Eli Lilly & Company, and Verve Therapeutics. All other authors have reported that they have no relationships relevant to the contents of this paper to disclose.
