# Supplementary material for: Mortality After Non–ST-Segment Elevation Myocardial Infarction: Impact of Left Ventricular Function and Coronary Artery Disease
Source: JACC Adv. 2025 Sep 16;4(10):102155. doi: 10.1016/j.jacadv.2025.102155 (PMC12476103; doi:10.1016/j.jacadv.2025.102155)
Supplement: Supplemental Data [file mmc1.docx]

| **Supplemental Table 1**. International Classification of Diseases codes, 10th revision (ICD-10) and Anatomical Therapeutic Chemical (ATC) codes used for baseline and outcome definition. | | |
| --- | --- | --- |
| *Condition* |  | *ICD-10 code* |
| **Cardiovascular death** |  | I10-I15, I20-I25, I46-I52 (except I51.4), I60-I69 (except I60, I62, I671, I675, I682), I70-I73, R960-R961 |
| Myocardial infarction |  | DI21 |
| Ischemic stroke |  | DI63, DI64 |
| Haemorrhagic stroke |  | DI60*, DI61*, DS06.6 |
| Hypertension |  | DI10*, DI15.1, DI15.8, DI15.9 |
| Peripheral artery disease |  | DI70, DI70.1−DI70.9. DI73.9 |
| Heart failure |  | DI11.0, DI13.0, DI13.2, DI50 |
| Atrial fibrillation/flutter |  | DI48 |
| Diabetes |  | DE10−DE14, DH36.0, DO24.0−24.3, DO24.5−24.9 |
| COPD |  | DJ40*-DJ47*, DJ60*-DJ67*, DJ68.4, DJ70.1, DJ70.3, *DJ84.1, DJ92.0, DJ96.1, DJ98.2, DJ98.3 |
| Percutaneous coronary intervention |  | KFNG02, KFNG05, KFNG96 |
| Coronary artery bypass grafting |  | KFNA−KFNE |
| *Drug* |  | *ATC code* |
| Aspirin |  | B01AC06, N02BA01 |
| Adenosine diphosphate-inhibitor |  | B01AC04, B01AC22, B01AC24 |
| Statin |  | C10AA |
| Direct oral anticoagulant |  | B01AF02, B01AE07, B01AF01 |
| Vitamin K-antagonist |  | B01AA03, B01AA04 |
| Beta-blocker |  | C07* |
| Angiotensin-converting enzyme inhibitor |  | C09A*, C09B* |
| Angiotensin-II receptor blocker |  | C09C*, C09D* |
| Mineralocorticoid receptor antagonist |  | C03DA* |
| Thiazide |  | C03AA*, C03AB* |
| Furosemide |  | C03DA* |
| Calcium channel blocker |  | C08C*, C08D* |
| Sodium-glucose transport protein 2 inhibitor |  | A10BK, A10BD15, A10BD16, A10BD19- A10BD21, A10BD23-A10BD25 |
| Insulin |  | A10A* |
| Non-insulin glucose-lowering drug |  | A10B* |

* Including subgroups

## **Supplemental Table 2**. Baseline characteristics of NSTEMI patients by extent of obstructive CAD

|  | 1VD  N = 4,581 | 2VD  N = 2,314 | 3VD  N = 1,875 |
| --- | --- | --- | --- |
| **Age in years, median (Q_1_-Q_3_)** | 65 (56, 74) | 69 (59, 77) | 72 (64, 80) |
| **Male sex, n (%)** | 3,154 (68.8) | 1,668 (72.1) | 1,378 (73.5) |
| **BMI (kg/m2), median (Q_1_-Q_3_)** | 27 (24, 30) | 27 (24, 30) | 27 (24, 30) |
| **Family history of premature IHD, n (%)** | 1,568 (34.2) | 804 (34.7) | 582 (31.0) |
| **Smoking, n (%)** | 1,419 (31.0) | 707 (30.6) | 459 (24.5) |
| **Comorbidities, n (%)** |  |  |  |
| Hypertension | 2,473 (54.0) | 1,424 (61.5) | 1,326 (70.7) |
| Heart failure before index CAG | 451 (9.8) | 391 (16.9) | 482 (25.7) |
| Previous ischemic stroke | 104 (2.3) | 106 (4.6) | 115 (6.1) |
| Previous haemorrhagic stroke | 16 (0.3) | 11 (0.5) | 9 (0.5) |
| Peripheral artery disease | 253 (5.5) | 207 (8.9) | 232 (12.4) |
| Atrial fibrillation/flutter | 478 (10.4) | 320 (13.8) | 320 (17.1) |
| Diabetes | 713 (15.6) | 518 (22.4) | 527 (28.1) |
| COPD | 437 (9.5) | 247 (10.7) | 182 (9.7) |
| **Left ventricular ejection fraction, n (%)** |  |  |  |
| >50% | 3,006 (65.6) | 1,246 (53.8) | 811 (43.3) |
| 41-50% | 1,053 (23.0) | 604 (26.1) | 506 (27.0) |
| ≤40% | 522 (11.4) | 464 (20.1) | 558 (29.8) |
| **Medication, n (%)** |  |  |  |
| Statin | 869 (19.0) | 610 (26.4) | 609 (32.5) |
| Beta-blocker | 566 (12.4) | 372 (16.1) | 329 (17.5) |
| ACE inhibitor | 588 (12.8) | 423 (18.3) | 394 (21.0) |
| ARB | 878 (19.2) | 519 (22.4) | 434 (23.1) |
| MRA | 71 (1.5) | 41 (1.8) | 40 (2.1) |
| SGLT2 inhibitor | 31 (0.7) | 21 (0.9) | 22 (1.2) |
| Furosemide | 248 (5.4) | 198 (8.6) | 188 (10.0) |
| Calcium blocker | 783 (17.1) | 478 (20.7) | 518 (27.6) |
| Thiazide | 408 (8.9) | 246 (10.6) | 232 (12.4) |
| Aspirin | 512 (11.2) | 395 (17.1) | 449 (23.9) |
| P2Y12 inhibitor | 141 (3.1) | 96 (4.1) | 106 (5.7) |
| VKA | 104 (2.3) | 82 (3.5) | 52 (2.8) |
| DOAC | 106 (2.3) | 65 (2.8) | 51 (2.7) |
| Insulin | 159 (3.5) | 132 (5.7) | 143 (7.6) |
| Non-insulin | 350 (7.6) | 269 (11.6) | 281 (15.0) |
| **Blood test values** |  |  |  |
| eGFR, mean (SD) | 80.6 (20.0) | 75.3 (22.5) | 70.8 (23.4) |
| Cholesterol, total (mmol/L), mean (SD) | 5.1 (4.4, 5.9) | 5.0 (4.2, 5.8) | 4.9 (4.0, 5.8) |
| LDL-cholesterol (mmol/L), mean (SD) | 3.1 (2.4, 3.8) | 3.0 (2.2, 3.8) | 2.9 (2.1, 3.8) |
| HDL-cholesterol (mmol/L), mean (SD) | 1.2 (1.0, 1.5) | 1.2 (1.0, 1.5) | 1.2 (1.0, 1.5) |
| Triglyceride (mmol/L), mean (SD) | 1.6 (1.1, 2.3) | 1.5 (1.1, 2.2) | 1.5 (1.1, 2.1) |
| HbA1c, median (Q_1_-Q_3_) | 39.0 (36.0, 42.0) | 40.0 (37.0, 45.0) | 40.0 (37.0, 47.0) |
| **Revascularisation day 0-30 from CAG, n (%)** |  |  |  |
| PCI, n (%) | 3,896 (85.0) | 1,839 (79.5) | 912 (48.6) |
| CABG, n (%) | 82 (1.8) | 288 (12.4) | 703 (37.5) |

**Caption:** Table S2 presents baseline characteristics of patients grouped by the extent of CAD. Comorbidities are registered until 30 days after the index CAG, except heart failure, which is only included until the day of the CAG.

**Abbreviations:** NSTEMI, non-ST-segment elevation myocardial infarction; CAD, coronary artery disease; VD, vessel disease; Q_1_-Q_3_, first and third quartiles; BMI, body mass index; IHD, ischemic heart disease; CAG, coronary angiography; COPD, chronic obstructive pulmonary disease; ACE inhibitor, angiotensin-converting-enzyme inhibitor; ARB, angiotensin receptor blocker; MRA, mineralocorticoid receptor antagonist; SGLT2 inhibitor, sodium-glucose transport protein 2 inhibitor; VKA, vitamin K antagonist; DOAC, direct oral anticoagulant; eGFR, estimated glomerular filtration rate; SD, standard deviation; LDL, low-density lipoprotein; HDL, high-density lipoprotein; PCI, percutaneous coronary intervention; CABG, coronary artery bypass graft.

| **Supplemental Table 3**. Baseline characteristics of NSTEMI patients by LVEF and extent of obstructive CAD  \|  \| LVEF >50%, 1VD  N = 3,006 \| LVEF >50%, 2VD  N = 1,246 \| LVEF >50%, 3VD  N = 811 \| LVEF 41-50%, 1VD  N = 1,053 \| LVEF 41-50%, 2VD  N = 604 \| LVEF 41-50%, 3 VD  N = 506 \| LVEF ≤40%, 1VD  N = 522 \| LVEF ≤40%, 2VD  N = 464 \| LVEF ≤40%, 3VD  N = 558 \| \| --- \| --- \| --- \| --- \| --- \| --- \| --- \| --- \| --- \| --- \| \| **Age in years, median (Q_1_-Q_3_)** \| 63 (54-72) \| 66 (58-75) \| 70 (61-78) \| 66 (57-76) \| 71 (60-79) \| 73 (65-80) \| 72 (64-80) \| 73 (65-80) \| 75 (67-81) \| \| **Male sex, n (%)** \| 2,100 (69.9) \| 914 (73.4) \| 622 (76.7) \| 737 (70.0) \| 445 (73.7) \| 364 (71.9) \| 317 (60.7) \| 309 (66.6) \| 392 (70.3) \| \| **BMI (kg/m2), median (Q_1_-Q_3_)** \| 27 (25-30) \| 27 (25-30) \| 27 (24-30) \| 27 (24-30) \| 27 (24-31) \| 27 (24-30) \| 27 (23-30) \| 26 (23-29) \| 26 (24-29) \| \| **Family history of premature IHD, n (%)** \| 1,098 (36.5) \| 482 (38.7) \| 284 (35.0) \| 335 (31.8) \| 214 (35.4) \| 170 (33.6) \| 135 (25.9) \| 108 (23.3) \| 128 (22.9) \| \| **Smoking, n (%)** \| 946 (31.5) \| 376 (30.2) \| 194 (23.9) \| 313 (29.7) \| 182 (30.1) \| 116 (22.9) \| 160 (30.7) \| 149 (32.1) \| 149 (26.7) \| \| **Comorbidities,**  **n (%)** \|  \|  \|  \|  \|  \|  \|  \|  \|  \| \| Hypertension \| 1,571 (52.3) \| 745 (59.8) \| 549 (67.7) \| 595 (56.5) \| 381 (63.1) \| 371 (73.3) \| 307 (58.8) \| 298 (64.2) \| 406 (72.8) \| \| Heart failure before index CAG \| 50 (1.7) \| 26 (2.1) \| 31 (3.8) \| 101 (9.6) \| 77 (12.7) \| 89 (17.6) \| 300 (57.5) \| 288 (62.1) \| 362 (64.9) \| \| Previous ischemic stroke \| 55 (1.8) \| 40 (3.2) \| 33 (4.1) \| 26 (2.5) \| 29 (4.8) \| 37 (7.3) \| 23 (4.4) \| 37 (8.0) \| 45 (8.1) \| \| Peripheral artery disease \| 126 (4.2) \| 79 (6.3) \| 70 (8.6) \| 78 (7.4) \| 57 (9.4) \| 62 (12.3) \| 49 (9.4) \| 71 (15.3) \| 100 (17.9) \| \| Atrial fibrillation/flutter \| 238 (7.9) \| 138 (11.1) \| 120 (14.8) \| 140 (13.3) \| 91 (15.1) \| 91 (18.0) \| 100 (19.2) \| 91 (19.6) \| 109 (19.5) \| \| Diabetes \| 406 (13.5) \| 243 (19.5) \| 169 (20.8) \| 178 (16.9) \| 142 (23.5) \| 140 (27.7) \| 129 (24.7) \| 133 (28.7) \| 218 (39.1) \| \| COPD \| 244 (8.1) \| 107 (8.6) \| 58 (7.2) \| 122 (11.6) \| 72 (11.9) \| 57 (11.3) \| 71 (13.6) \| 68 (14.7) \| 67 (12.0) \| \| **Medication, n (%)** \|  \|  \|  \|  \|  \|  \|  \|  \|  \| \| Statin \| 544 (18.1) \| 313 (25.1) \| 235 (29.0) \| 193 (18.3) \| 143 (23.7) \| 156 (30.8) \| 132 (25.3) \| 154 (33.2) \| 218 (39.1) \| \| Beta-blocker \| 330 (11.0) \| 162 (13.0) \| 125 (15.4) \| 142 (13.5) \| 105 (17.4) \| 93 (18.4) \| 94 (18.0) \| 105 (22.6) \| 111 (19.9) \| \| ACE inhibitor \| 369 (12.3) \| 210 (16.9) \| 151 (18.6) \| 128 (12.2) \| 119 (19.7) \| 113 (22.3) \| 91 (17.4) \| 94 (20.3) \| 130 (23.3) \| \| ARB \| 543 (18.1) \| 278 (22.3) \| 187 (23.1) \| 220 (20.9) \| 142 (23.5) \| 114 (22.5) \| 115 (22.0) \| 99 (21.3) \| 133 (23.8) \| \| MRA \| 29 (1.0) \| 17 (1.4) \| 12 (1.5) \| 22 (2.1) \| 12 (2.0) \| 11 (2.2) \| 20 (3.8) \| 12 (2.6) \| 17 (3.0) \| \| SGLT2 inhibitor \| 17 (0.6) \| 10 (0.8) \| <5 \| 11 (1.0) \| <5 \| <5 \| 8 (1.7) \| 12 (2.2) \| 12 (2.2) \| \| Furosemide \| 119 (4.0) \| 65 (5.2) \| 56 (6.9) \| 69 (6.6) \| 69 (11.4) \| 57 (11.3) \| 60 (11.5) \| 64 (13.8) \| 75 (13.4) \| \| Calcium blocker \| 480 (16.0) \| 245 (19.7) \| 224 (27.6) \| 188 (17.9) \| 128 (21.2) \| 133 (26.3) \| 115 (22.0) \| 105 (22.6) \| 161 (28.9) \| \| Thiazide \| 265 (8.8) \| 139 (11.2) \| 94 (11.6) \| 85 (8.1) \| 54 (8.9) \| 59 (11.7) \| 58 (11.1) \| 53 (11.4) \| 79 (14.2) \| \| Aspirin \| 292 (9.7) \| 176 (14.1) \| 168 (20.7) \| 137 (13.0) \| 104 (17.2) \| 105 (20.8) \| 83 (15.9) \| 115 (24.8) \| 176 (31.5) \| \| P2Y12 inhibitor \| 81 (2.7) \| 40 (3.2) \| 37 (4.6) \| 40 (3.8) \| 33 (5.5) \| 38 (7.5) \| 20 (3.8) \| 23 (5.0) \| 31 (5.6) \| \| VKA \| 45 (1.5) \| 39 (3.1) \| 12 (1.5) \| 39 (3.7) \| 18 (3.0) \| 18 (3.6) \| 20 (3.8) \| 25 (5.4) \| 22 (3.9) \| \| DOAC \| 48 (1.6) \| 29 (2.3) \| 23 (2.8) \| 36 (3.4) \| 19 (3.1) \| 15 (3.0) \| 22 (4.2) \| 17 (3.7) \| 13 (2.3) \| \| Insulin \| 82 (2.7) \| 54 (4.3) \| 37 (4.6) \| 46 (4.4) \| 35 (5.8) \| 40 (7.9) \| 31 (5.9) \| 43 (9.3) \| 66 (11.8) \| \| Non-insulin \| 202 (6.7) \| 123 (9.9) \| 88 (10.9) \| 90 (8.5) \| 78 (12.9) \| 76 (15.0) \| 58 (11.1) \| 68 (14.7) \| 117 (21.0) \| \| **Blood test values** \|  \|  \|  \|  \|  \|  \|  \|  \|  \| \| eGFR, mean (SD) \| 83.0 (18.7) \| 79.4 (20.2) \| 75.7 (21.8) \| 77.9 (20.8) \| 73.8 (22.7) \| 69.9 (23.6) \| 72.1 (22.3) \| 66.5 (25.2) \| 64.3 (23.7) \| \| Cholesterol, total (mmol/L),  median (Q_1_-Q_3_) \| 5.2 (4.5-5.9) \| 5.2 (4.3-6.0) \| 5.1 (4.3-5.9) \| 5.0 (4.4-5.9) \| 4.9 (4.3-5.8) \| 4.9 (3.9-5.9) \| 4.8 (4.0-5.7) \| 4.6 (3.8-5.5) \| 4.5 (3.7-5.4) \| \| LDL-cholesterol (mmol/L),  median (Q_1_-Q_3_) \| 3.2 (2.5-3.8) \| 3.2 (2.3-3.9) \| 3.1 (2.4-3.9) \| 3.0 (2.3-3.8) \| 3.0 (2.2-3.8) \| 2.9 (2.1-3.8) \| 2.8 (2.2-3.6) \| 2.6 (1.9-3.4) \| 2.5 (1.9-3.4) \| \| HDL-cholesterol (mmol/L),  median (Q_1_-Q_3_) \| 1.2 (1.0-1.5) \| 1.2 (1.0-1.4) \| 1.2 (1.0-1.4) \| 1.2 (1.0-1.5) \| 1.2 (1.0-1.5) \| 1.2 (1.0-1.5) \| 1.2 (1.0-1.6) \| 1.2 (1.0-1.5) \| 1.2 (1.0-1.5) \| \| Triglyceride (mmol/L),  median (Q_1_-Q_3_) \| 1.6 (1.1-2.4) \| 1.6 (1.1-2.4) \| 1.6 (1.1-2.2) \| 1.5 (1.1-2.3) \| 1.5 (1.0-2.1) \| 1.5 (1.1-2.1) \| 1.4 (1.0-1.9) \| 1.4 (1.0-2.0) \| 1.4 (1.0-1.9) \| \| HbA1c,  median (Q_1_-Q_3_) \| 38 (36-42) \| 39 (36-44) \| 40 (37- 44) \| 39 (36-42) \| 40 (37-45) \| 40 (37-47) \| 40 (37-46) \| 41 (38-49) \| 42 (38-52) \| \| **Revascularization day 0-30 after CAG, n (%)** \|  \|  \|  \|  \|  \|  \|  \|  \|  \| \| PCI \| 2,575 (85.7) \| 1,003 (80.5) \| 383 (47.2) \| 890 (84.5) \| 482 (79.8) \| 246 (48.6) \| 431 (82.6) \| 354 (76.3) \| 283 (50.7) \| \| CABG \| 58 (1.9) \| 166 (13.3) \| 347 (42.8) \| 15 (1.4) \| 78 (12.9) \| 185 (36.6) \| 9 (1.7) \| 44 (9.5) \| 171 (30.6) \| |
| --- | --- | --- | --- | --- | --- | --- | --- | --- | --- | --- | --- | --- | --- | --- | --- | --- | --- | --- | --- | --- | --- | --- | --- | --- | --- | --- | --- | --- | --- | --- | --- | --- | --- | --- | --- | --- | --- | --- | --- | --- | --- | --- | --- | --- | --- | --- | --- | --- | --- | --- | --- | --- | --- | --- | --- | --- | --- | --- | --- | --- | --- | --- | --- | --- | --- | --- | --- | --- | --- | --- | --- | --- | --- | --- | --- | --- | --- | --- | --- | --- | --- | --- | --- | --- | --- | --- | --- | --- | --- | --- | --- | --- | --- | --- | --- | --- | --- | --- | --- | --- | --- | --- | --- | --- | --- | --- | --- | --- | --- | --- | --- | --- | --- | --- | --- | --- | --- | --- | --- | --- | --- | --- | --- | --- | --- | --- | --- | --- | --- | --- | --- | --- | --- | --- | --- | --- | --- | --- | --- | --- | --- | --- | --- | --- | --- | --- | --- | --- | --- | --- | --- | --- | --- | --- | --- | --- | --- | --- | --- | --- | --- | --- | --- | --- | --- | --- | --- | --- | --- | --- | --- | --- | --- | --- | --- | --- | --- | --- | --- | --- | --- | --- | --- | --- | --- | --- | --- | --- | --- | --- | --- | --- | --- | --- | --- | --- | --- | --- | --- | --- | --- | --- | --- | --- | --- | --- | --- | --- | --- | --- | --- | --- | --- | --- | --- | --- | --- | --- | --- | --- | --- | --- | --- | --- | --- | --- | --- | --- | --- | --- | --- | --- | --- | --- | --- | --- | --- | --- | --- | --- | --- | --- | --- | --- | --- | --- | --- | --- | --- | --- | --- | --- | --- | --- | --- | --- | --- | --- | --- | --- | --- | --- | --- | --- | --- | --- | --- | --- | --- | --- | --- | --- | --- | --- | --- | --- | --- | --- | --- | --- | --- | --- | --- | --- | --- | --- | --- | --- | --- | --- | --- | --- | --- | --- | --- | --- | --- | --- | --- | --- | --- | --- | --- | --- | --- | --- | --- | --- | --- | --- | --- | --- | --- | --- | --- | --- | --- | --- | --- | --- | --- | --- | --- | --- | --- | --- | --- | --- | --- | --- | --- | --- | --- | --- | --- | --- | --- | --- | --- | --- | --- | --- | --- | --- | --- | --- | --- | --- | --- | --- | --- | --- | --- | --- | --- | --- | --- | --- | --- | --- | --- | --- | --- | --- | --- | --- | --- | --- | --- | --- | --- | --- | --- | --- | --- | --- | --- | --- | --- | --- | --- | --- | --- | --- | --- | --- | --- | --- | --- | --- | --- | --- | --- | --- | --- | --- | --- | --- | --- | --- |

**Caption:** Table S3 presents baseline characteristics of patients grouped by LVEF and extent of obstructive CAD. Comorbidities are registered until 30 days after the index CAG, except heart failure, which is only included until the day of the CAG.

**Abbreviations:** ACE inhibitor, angiotensin-converting-enzyme inhibitor; ARB, angiotensin receptor blocker; BMI, body mass index; CABG, coronary artery bypass graft; CAG, coronary angiography; COPD, chronic obstructive pulmonary disease; DOAC, direct oral anticoagulant; eGFR, estimated glomerular filtration rate; HDL, high-density lipoprotein; IHD, ischemic heart disease; LDL, low-density lipoprotein; LVEF, left ventricular ejection fraction; MRA, mineralocorticoid receptor antagonist; NSTEMI, non-ST-segment elevation myocardial infarction; PCI, percutaneous coronary intervention; Q_1_-Q_3_, first and third quartiles; SD, standard deviation; SGLT2 inhibitor, sodium-glucose transport protein 2 inhibitor; VD, vessel disease; VKA, vitamin K antagonist.

##

## **Supplemental Table 4.** Baseline characteristics of NSTEMI patients by 30-day revascularization

|  | Revascularization  N = 7,612 | No revascularization  N = 1,158 |
| --- | --- | --- |
| **Age in years, median (Q_1_-Q_3_)** | 67 (58, 75) | 71 (61, 80) |
| **Male sex, n (%)** | 5,544 (72.8) | 656 (56.6) |
| **BMI (kg/m2), median (Q_1_-Q_3_)** | 27 (24-30) | 26 (23-29) |
| **Family history of premature IHD, n (%)** | 2,622 (34.4) | 332 (28.7) |
| **Smoking, n (%)** | 2,289 (30.1) | 296 (25.6) |
| **Comorbidities, n (%)** |  |  |
| Hypertension | 4,444 (58.4) | 779 (67.3) |
| Heart failure before index CAG | 1,081 (14.2) | 243 (21.0) |
| Previous ischemic stroke | 252 (3.3) | 73 (6.3) |
| Previous hemorrhagic stroke | 11 (0.5) | 7 (0.3) |
| Peripheral artery disease | 538 (7.1) | 154 (13.3) |
| Atrial fibrillation/flutter | 911 (12.0) | 207 (17.9) |
| Diabetes | 1,464 (19.2) | 294 (25.4) |
| COPD | 718 (9.4) | 148 (12.8) |
| **Coronary artery disease, n (%)** |  |  |
| 1VD | 3,972 (52.2) | 609 (52.6) |
| 2VD | 2,088 (27.4) | 226 (19.5) |
| 3VD | 1,552 (20.4) | 323 (27.9) |
| **Medication, n (%)** |  |  |
| Statin | 1,752 (23.0) | 336 (29.0) |
| Beta-blocker | 1,039 (13.6) | 228 (19.7) |
| ACE inhibitor | 1,174 (15.4) | 231 (19.9) |
| ARB | 1,589 (20.9) | 242 (20.9) |
| MRA | 123 (1.6) | 29 (2.5) |
| SGLT2 inhibitor | 67 (0.9) | 7 (0.6) |
| Furosemide | 494 (6.5) | 140 (12.1) |
| Calcium blocker | 1,501 (19.7) | 278 (24.0) |
| Thiazide | 712 (9.4) | 174 (15.0) |
| Aspirin | 1,093 (14.4) | 263 (22.7) |
| P2Y12 inhibitor | 273 (3.6) | 70 (6.0) |
| VKA | 186 (2.4) | 52 (4.5) |
| DOAC | 174 (2.3) | 48 (4.1) |
| Insulin | 353 (4.6) | 81 (7.0) |
| Non-insulin | 745 (9.8) | 155 (13.4) |
| **Blood test values** |  |  |
| eGFR, mean (SD) | 77.8 (21.4) | 72.5 (23.5) |
| Cholesterol, total (mmol/L), mean (SD) | 5.1 (4.3, 5.9) | 4.7 (3.9, 5.6) |
| LDL-cholesterol (mmol/L), mean (SD) | 3.1 (2.3, 3.8) | 2.6 (2.0, 3.4) |
| HDL-cholesterol (mmol/L), mean (SD) | 1.2 (1.0, 1.5) | 1.3 (1.1, 1.6) |
| Triglyceride (mmol/L), mean (SD) | 1.6 (1.1, 2.3) | 1.3 (1.0, 1.9) |
| HbA1c, median (Q_1_-Q_3_) | 39.0 (36.0, 44.0) | 40.0 (36.0, 45.0) |
| **Revascularization day 0-30 after CAG, n (%)** |  |  |
| PCI | 6,647 (87.3) | 0 (0.0) |
| CABG | 1,073 (14.1) | 0 (0.0) |

**Caption:** Table S4 presents baseline characteristics of patients grouped by whether they receive revascularization within 30 days after the CAG. Comorbidities are registered until 30 days after the index CAG, except heart failure, which is only included until the day of the CAG.

**Abbreviations:** ACE inhibitor, angiotensin-converting-enzyme inhibitor; ARB, angiotensin receptor blocker; BMI, body mass index; CABG, coronary artery bypass graft; CAG, coronary angiography; COPD, chronic obstructive pulmonary disease; DOAC, direct oral anticoagulant; eGFR, estimated glomerular filtration rate; HDL, high-density lipoprotein; IHD, ischemic heart disease; LDL, low-density lipoprotein; LVEF, left ventricular ejection fraction; MRA, mineralocorticoid receptor antagonist; NSTEMI, non-ST-segment elevation myocardial infarction; PCI, percutaneous coronary intervention; Q_1_-Q_3_, first and third quartiles; SD, standard deviation; SGLT2 inhibitor, sodium-glucose transport protein 2 inhibitor; VD, vessel disease; VKA, vitamin K antagonist.

## **Supplemental Table 5**. Number of NSTEMI patients with redeemed prescriptions 0-180 days after CAG grouped by LVEF

|  | LVEF >50%  N=5,063 | LVEF 41-50%  N=2,163 | LVEF ≤40%  N=1,544 |
| --- | --- | --- | --- |
| **Medication, n (%)** |  |  |  |
| Statin | 4,846 (95.7) | 2,006 (92.7) | 1,316 (85.2) |
| Beta-blocker | 3,377 (66.7) | 1,573 (72.7) | 1,350 (87.4) |
| ACE inhibitor | 1,274 (25.2) | 767 (35.5) | 1,055 (68.3) |
| ARB | 1,141 (22.5) | 533 (24.6) | 373 (24.2) |
| MRA | 140 (2.8) | 147 (6.8) | 465 (30.1) |
| SGLT2 inhibitor | 86 (1.7) | 47 (2.2) | 71 (4.6) |
| Furosemide | 689 (13.6) | 577 (26.7) | 1,017 (65.9) |
| Calcium blocker | 1,621 (32.0) | 626 (28.9) | 289 (18.7) |
| Thiazide | 545 (10.8) | 195 (9.0) | 113 (7.3) |
| Aspirin | 4,801 (94.8) | 1,985 (91.8) | 1,357 (87.9) |
| P2Y12 inhibitor | 4,755 (93.9) | 1,968 (91.0) | 1,374 (89.0) |
| VKA | 197 (3.9) | 132 (6.1) | 161 (10.4) |
| DOAC | 345 (6.8) | 229 (10.6) | 211 (13.7) |
| Insulin | 230 (4.5) | 158 (7.3) | 197 (12.8) |
| Non-insulin | 582 (11.5) | 311 (14.4) | 303 (19.6) |

**Caption:** Table S5 depicts the number and percentage of NSTEMI patients grouped by LVEF who redeemed prescriptions for different types of medication between 0 and 180 days after the index CAG.

**Abbreviations:** NSTEMI, non-ST-segment elevation myocardial infarction; CAG, coronary angiography; LVEF, left ventricular ejection fraction; ACE inhibitor, angiotensin-converting-enzyme inhibitor; ARB, angiotensin receptor blocker; MRA, mineralocorticoid receptor antagonist; SGLT2 inhibitor, sodium-glucose transport protein 2 inhibitor; VKA, vitamin K antagonist; DOAC, direct oral anticoagulant.

## **Supplemental Table 6.** Number of NSTEMI patients with redeemed prescriptions 0-180 days after CAG grouped by the extent of CAD

|  | 1VD  N=4,581 | 2VD  N=2,314 | 3VD  N=1,875 |
| --- | --- | --- | --- |
| **Medication, n (%)** |  |  |  |
| Statin | 4,318 (94.3) | 2,164 (93.5) | 1,686 (89.9) |
| Beta-blocker | 3,083 (67.3) | 1,754 (75.8) | 1,463 (78.0) |
| ACE inhibitor | 1,396 (30.5) | 869 (37.6) | 831 (44.3) |
| ARB | 1,040 (22.7) | 579 (25.0) | 428 (22.8) |
| MRA | 284 (6.2) | 216 (9.3) | 252 (13.4) |
| SGLT2 inhibitor | 78 (1.7) | 60 (2.6) | 66 (3.5) |
| Furosemide | 724 (15.8) | 688 (29.7) | 871 (46.5) |
| Calcium blocker | 1,235 (27.0) | 704 (30.4) | 597 (31.8) |
| Thiazide | 410 (9.0) | 249 (10.8) | 194 (10.3) |
| Aspirin | 4,304 (94.0) | 2,144 (92.7) | 1,695 (90.4) |
| P2Y12 inhibitor | 4,364 (95.3) | 2,157 (93.2) | 1,576 (84.1) |
| VKA | 193 (4.2) | 149 (6.4) | 148 (7.9) |
| DOAC | 337 (7.4) | 216 (9.3) | 232 (12.4) |
| Insulin | 197 (4.3) | 184 (8.0) | 204 (10.9) |
| Non-insulin | 496 (10.8) | 347 (15.0) | 353 (18.8) |

**Caption:** Table S6 depicts the number and percentage of NSTEMI patients grouped by the extent of CAD who redeemed prescriptions for different types of medication between 0 and 180 days after the index CAG.

**Abbreviations:** NSTEMI, non-ST-segment elevation myocardial infarction; CAG, coronary angiography; CAD, coronary artery disease; ACE inhibitor, angiotensin-converting-enzyme inhibitor; ARB, angiotensin receptor blocker; MRA, mineralocorticoid receptor antagonist; SGLT2 inhibitor, sodium-glucose transport protein 2 inhibitor; VKA, vitamin K antagonist; DOAC, direct oral anticoagulant.

|  | Patients | Deaths | Five-year CIP | Unadjusted HR | Adjusted HR* using LVEF >50% and 1VD as ref | Adjusted HR* using 1VD as ref |
| --- | --- | --- | --- | --- | --- | --- |
| **LVEF >50%** |  |  |  |  |  |  |
| 1VD | 2991 | 206 | 8.9% (7.8-10.1) | Ref | Ref | Ref |
| 2VD | 1240 | 130 | 12.8% (10.9-15.1) | 1.52 (1.22-1.90) | 1.20 (0.96-1.49) | 1.18 (0.94-1.47) |
| 3VD | 802 | 130 | 19.8% (16.9-23.2) | 2.42 (1.94-3.01) | 1.57 (1.25-1.96) | 1.51 (1.20-1.89) |
| **LVEF 41-50%** |  |  |  |  |  |  |
| 1VD | 1032 | 131 | 16.2% (13.8-19.1) | 1.94 (1.56.2.41) | 1.52 (1.22-1.89) | Ref |
| 2VD | 589 | 117 | 25.4% (21.6-29.8) | 3.02 (2.41-3.79) | 1.87 (1.49-2.36) | 1.19 (0.93-1.54) |
| 3VD | 487 | 122 | 29.1% (24.9-33.8) | 3.92 (3.13-4.90) | 2.09 (1.66-2.63) | 1.32 (1.03-1.70) |
| **LVEF ≤40 %** |  |  |  |  |  |  |
| 1VD | 493 | 124 | 31.8% (27.2-36.9) | 4.07 (3.26-5.09) | 2.39 (1.91-3.00) | Ref |
| 2VD | 437 | 145 | 37.4% (32.7-42.6) | 5.70 (4.61-7.05) | 3.11 (2.50-3.86) | 1.31 (1.03-1.67) |
| 3VD | 527 | 202 | 44.9% (40.3-49.9) | 6.74 (5.55-8.19) | 3.03 (2.47-3.70) | 1.33 (1.06-1.68) |

## **Supplemental Table 7.** Five-year mortality among patients with first-time NSTEMI grouped by LVEF and extent of obstructive CAD, excluding those with a heart failure diagnosis dated >30 days before CAG

Patients and deaths are listed as counts, CIP as percentages with 95% CI, and HR as ratios with 95% CI.
**Caption:** Table S7 presents a sensitivity analysis of mortality grouped by LVEF and the extent of CAD, excluding those with a heart failure diagnosis dated >30 days before the index CAG. Adjusted HRs are shown both with LVEF >50% and 1VD as a reference and with 1VD as a reference within each group of LVEF. An increasing 5-year CIP was observed with a decreasing LVEF and increasing extent of CAD.
* Adjusted for sex, age, hypertension, previous ischemic stroke, peripheral artery disease, atrial fibrillation, smoking, and diabetes.

**Abbreviations:** NSTEMI, non-ST-segment elevation myocardial infarction; LVEF, left ventricular ejection fraction; CAD, coronary artery disease; CAG, coronary angiography; CIP, cumulative incidence proportion; HR, hazard ratio; VD, vessel disease; CI, confidence interval.

## **Supplemental Table 8.** Five-year mortality after first-time NSTEMI grouped by LVEF and extent of obstructive CAD in patients who underwent revascularization within 30 days after CAG

|  | Patients | Deaths | Five-year CIP | Unadjusted HR | Adjusted HR* using LVEF >50% and 1VD as ref | Adjusted HR* using 1VD as ref |
| --- | --- | --- | --- | --- | --- | --- |
| **LVEF >50%** |  |  |  |  |  |  |
| 1VD | 2,630 | 170 | 8.3% (7.2-9.7) | Ref | Ref | Ref |
| 2VD | 1,146 | 106 | 11.5% (9.5-13.7) | 1.42 (1.12-1.81) | 1.12 (0.88-1.43) | 1.10 (0.86-1.40) |
| 3VD | 700 | 87 | 15.5% (12.7-18.9) | 1.96 (1.51-2.54) | 1.29 (0.99-1.68) | 1.23 (1.95-1.61) |
| **LVEF 41-50%** |  |  |  |  |  |  |
| 1VD | 903 | 107 | 15.2% (12.7-18.2) | 1.91 (1.50-2.44) | 1.51 (1.19-1.93) | Ref |
| 2VD | 551 | 106 | 24.8% (20.9-29.3) | 3.11 (2.44-3.96) | 1.90 (1.49-2.44) | 1.22 (0.93-1.60) |
| 3VD | 412 | 74 | 21.8% (17.6-26.7) | 2.87 (2.19-3.77) | 1.57 (1.19-2.08) | 1.00 (1.74-1.36) |
| **LVEF ≤40 %** |  |  |  |  |  |  |
| 1VD | 439 | 114 | 32.7% (27.8-38.1) | 4.51 (3.56-5.72) | 2.57 (2.02-3.28) | Ref |
| 2VD | 391 | 121 | 35.2% (30.3-40.7) | 5.65 (4.47-7.13) | 2.98 (2.35-3.78) | 1.16 (0.90-1.50) |
| 3VD | 440 | 148 | 39.8% (34.9-45.2) | 6.04 (4.85-7.54) | 2.74 (2.18-3.44) | 1.10 (0.86-1.41) |

Patients and deaths are listed as counts, CIP as percentages with 95% CI, and HR as ratios with 95% CI.
**Caption:** Table S8 presents mortality grouped by LVEF and the extent of obstructive CAD in patients who underwent revascularization within 30 days.
Adjusted HRs are shown both with LVEF >50% and 1VD as a reference and with 1VD as a reference within each group of LVEF. An increasing 5-year CIP was observed with a decreasing LVEF and increasing extent of CAD.
*Adjusted for sex, age, hypertension, previous ischemic stroke, peripheral artery disease, atrial fibrillation, smoking, and diabetes.
**Abbreviations:** CAD, coronary artery disease; CAG, coronary angiography; CI, confidence interval; CIP, cumulative incidence proportion; HR, hazard ratio; LVEF, left ventricular ejection fraction; NSTEMI, non-ST-segment elevation myocardial infarction; VD, vessel disease.

## **Supplemental Table 9.** Five-year mortality after first-time NSTEMI grouped by LVEF and extent of obstructive CAD in patients who did *not* undergo revascularization within 30 days after CAG

|  | Patients | Deaths | Five-year CIP | Unadjusted HR | Adjusted HR* using LVEF >50% and 1VD as ref | Adjusted HR* using 1VD as ref |
| --- | --- | --- | --- | --- | --- | --- |
| **LVEF >50%** |  |  |  |  |  |  |
| 1VD | 376 | 44 | 14.5% (11.0-19.4) | Ref | Ref | Ref |
| 2VD | 100 | 27 | 30.3% (21.8-41.2) | 2.49 (1.54-4.02) | 1.71 (1.05-2.78) | 1.61 (0.97-2.66) |
| 3VD | 111 | 49 | 48.3% (38.9-58.6) | 4.13 (2.75-6.02) | 2.53 (1.67-3.84) | 2.42 (1.56-3.76) |
| **LVEF 41-50%** |  |  |  |  |  |  |
| 1VD | 150 | 34 | 27.6% (20.3-36.9) | 2.14 (1.36-3.34) | 1.73 (1.10-2.71) | Ref |
| 2VD | 53 | 21 | 45.6% (32.2-61.4) | 4.00 (2.38-6.72) | 2.31 (1.36-3.92) | 1.28 (0.73-2.24) |
| 3VD | 94 | 55 | 62.5% (52.2-72.9) | 6.55 (4.40-9.73) | 3.30 (2.18-4.99) | 1.80 (1.14-2.85) |
| **LVEF ≤40 %** |  |  |  |  |  |  |
| 1VD | 83 | 27 | 39.0% (28.3-52.2) | 3.13 (1.94-5.06) | 2.00 (1.23-3.24) | Ref |
| 2VD | 73 | 39 | 56.7% (45.1-68.9) | 6.16 (4.00-9.48) | 4.42 (2.84-6.88) | 2.29 (1.38-3.81) |
| 3VD | 118 | 74 | 69.3% (60.0-78.2) | 7.70 (5.30-11.20) | 3.81 (2.56-5.66) | 2.12 (1.31-3.45) |

Patients and deaths are listed as counts, CIP as percentages with 95% CI, and HR as ratios with 95% CI.
**Caption:** Table S9 presents mortality grouped by LVEF and the extent of obstructive CAD in patients who did not undergo revascularization within 30 days.
Adjusted HRs are shown both with LVEF >50% and 1VD as a reference and with 1VD as a reference within each group of LVEF. An increasing 5-year CIP was observed with a decreasing LVEF and increasing extent of CAD.
*Adjusted for sex, age, hypertension, previous ischemic stroke, peripheral artery disease, atrial fibrillation, smoking, and diabetes.
**Abbreviations:** CAD, coronary artery disease; CAG, coronary angiography; CI, confidence interval; CIP, cumulative incidence proportion; HR, hazard ratio; LVEF, left ventricular ejection fraction; NSTEMI, non-ST-segment elevation myocardial infarction; VD, vessel disease.

## **Supplemental Table 10**. Five-year mortality after first-time NSTEMI by LVEF and extent of obstructive CAD including 30-day revascularization in the multivariable regression model

|  | Patients | Deaths | Adjusted HR* using LVEF >50% and 1VD as ref | Adjusted HR* using 1VD as ref |
| --- | --- | --- | --- | --- |
| **LVEF >50%** |  |  |  |  |
| 1VD | 3,006 | 214 | Ref | Ref |
| 2VD | 1,246 | 133 | 1.24 (0.99-1.54) | 1.21 (0.97-1.50) |
| 3VD | 811 | 136 | 1.58 (1.27-1.96) | 1.50 (1.20-1.87) |
| **LVEF 41-50%** |  |  |  |  |
| 1VD | 1,053 | 141 | 1.57 (1.26-1.94) | Ref |
| 2VD | 604 | 127 | 2.01 (1.61-2.52) | 1.25 (0.98-1.60) |
| 3VD | 506 | 129 | 1.99 (1.59-2.49) | 1.21 (0.94-1.54) |
| **LVEF ≤40 %** |  |  |  |  |
| 1VD | 522 | 141 | 2.42 (1.95-3.00) | Ref |
| 2VD | 464 | 160 | 3.27 (2.65-4.03) | 1.33 (1.06-1.68) |
| 3VD | 558 | 222 | 2.99 (2.46-3.64) | 1.29 (1.04-1.60) |

Values are listed as numbers or percentages/ratios with 95% CI.

**Caption:** Table S10 presents mortality grouped by LVEF and the extent of obstructive CAD including 30-day revascularization with PCI or CABG in the multivariable regression model.
Adjusted HRs are shown both with LVEF >50% and 1VD as a reference and with 1VD as a reference within each group of LVEF. An increasing 5-year CIP was observed with a decreasing LVEF and increasing extent of CAD.
*Adjusted for sex, age, hypertension, previous ischemic stroke, peripheral artery disease, atrial fibrillation, smoking, and diabetes.

**Abbreviations:** CABG, coronary artery bypass graft; CAD, coronary artery disease; CI, confidence interval; HR, hazard ratio; LVEF, left ventricular ejection fraction; NSTEMI, non-ST-segment elevation myocardial infarction; PCI, percutaneous coronary intervention; VD, vessel disease.

## **Supplemental Table 11**. Five-year all-cause and cardiovascular mortality by LVEF and extent of obstructive CAD

|  | Patients | | Events | | Five-year CIP* | | Unadjusted HR | | Age- and sex-adjusted HR | | Adjusted HR† | |
| --- | --- | --- | --- | --- | --- | --- | --- | --- | --- | --- | --- | --- |
| ***All-cause***  ***mortality***  **LVEF** |  | |  | |  | |  | |  | |  | |
| LVEF >50% | 5,063 | | 483 | | 11.9% (11.0-13.0) | | Ref | | Ref | | Ref | |
| LVEF 41-50% | 2,163 | | 397 | | 22.9% (20.9-25.0) | | 2.03 (1.78-2.32) | | 1.65 (1.44-1.88) | | 1.49 (1.31-1.71) | |
| LVEF ≤40% | 1,544 | | 523 | | 39.8% (37.1-42.7) | | 4.16 (3.68-4.17) | | 2.82 (2.49-3.20) | | 2.32 (2.04-2.65) | |
|  |  | |  | |  | |  | |  | |  | |
| **CAD extent** |  | |  | |  | |  | |  | |  | |
| 1VD | 4,581 | | 496 | | 13.8% (12.7-15.0) | | Ref | | Ref | | Ref | |
| 2VD | | 2,314 | | 420 | | 21.8% (20.0-23.8) | | 1.72 (1.51-1.95) | | 1.43 (1.26-1.63) | | 1.24 (1.09-1.41) |
| 3VD | | 1,875 | | 487 | | 30.7% (28.4-33.1) | | 2.56 (2.26-2.90) | | 1.79 (1.57-1.63) | | 1.35 (1.19-1.54) |
|  | |  | |  | |  | |  | |  | |  |
| ***Cardiovascular mortality***  **LVEF** | |  | |  | |  | |  | |  | |  |
| LVEF >50% | | 5,063 | | 136 | | 3.3% (2.8-3.9) | | Ref | | Ref | | Ref |
| LVEF 41-50% | | 2,163 | | 133 | | 7.8% (6.5-9.1) | | 2.41 (1.89-3.05) | | 1.91 (1.50-2.43) | | 1.67 (1.31-2.13) |
| LVEF ≤40% | | 1,544 | | 229 | | 17.1% (15.1-19.2) | | 6.42 (5.19-7.93) | | 4.21 (3.39-5.22) | | 3.23 (2.59-4.03) |
|  | |  | |  | |  | |  | |  | |  |
| **CAD extent** | |  | |  | |  | |  | |  | |  |
| 1VD | | 4,581 | | 132 | | 3.7% (3.1-4.3) | | Ref | | Ref | | Ref |
| 2VD | | 2,314 | | 152 | | 7.7% (6.6-9.0) | | 2.33 (1.85-2.94) | | 1.93 (1.53-2.44) | | 1.62 (1.28-2.05) |
| 3VD | | 1,875 | | 214 | | 13.3% (11.7-15.1) | | 4.21 (3.39-5.23) | | 2.89 (2.32-3.60) | | 2.08 (1.66-2.61) |

Values are listed as numbers or percentages/ratios with 95% CI.

**Caption:** Table S11 depicts a 5-year all-cause mortality risk and cardiovascular mortality, grouped by LVEF and extent of obstructive CAD. An increasing CIP and adjusted HR were found with decreasing LVEF and increasing CAD extent for both outcomes.
*CIP for cardiovascular mortality adjusted for the competing risk of non-cardiovascular mortality.

†Adjusted for sex, age, hypertension, previous ischemic stroke, peripheral artery disease, atrial fibrillation, smoking, and diabetes. Additionally, CAD extent was adjusted for LVEF, and LVEF was adjusted for CAD extent.

**Abbreviations:** CAD, coronary artery disease; CI, confidence interval; CIP, cumulative incidence proportion; HR, hazard ratio; LVEF, left ventricular ejection fraction; VD, vessel disease.

## **Supplemental Table 12. Five-year mortality by sex, age, and LVEF in patients with NSTEMI**

|  | Patients | Events | Five-year CIP | Risk difference | Unadjusted HR | Adjusted HR* |
| --- | --- | --- | --- | --- | --- | --- |
| **Male sex** |  |  |  |  |  |  |
| LVEF >50% | 3,636 | 303 | 10.5% (9.4-11.7) | Ref | Ref | Ref |
| LVEF 41-50% | 1,546 | 262 | 21.3% (19.0-23.8) | 10.8% (8.2-13.4) | 2.15 (1.82-2.54) | 1.55 (1.31-1.83) |
| LVEF ≤40% | 1,018 | 341 | 39.4% (36.1-42.8) | 28.9% (25.3-32.4) | 4.74 (4.06-5.54) | 2.43 (2.07-2.87) |
| **Female sex** |  |  |  |  |  |  |
| LVEF >50% | 1,427 | 180 | 15.6% (13.6-17.9) | Ref | Ref | Ref |
| LVEF 41-50% | 617 | 135 | 26.8% (23.1-31.1) | 11.2% (6.7-15.8) | 1.81 (1.45-2.27) | 1.41 (1.13-1.77) |
| LVEF ≤40% | 526 | 182 | 40.7% (36.2-45.6) | 25.1% (19.9-30.3) | 3.15 (2.57-3.88) | 2.15 (1.74-2.67) |
|  |  |  |  |  |  |  |
| **Age 18-64** |  |  |  |  |  |  |
| LVEF >50% | 2,499 | 74 | 3.7% (3.0-4.7) | Ref | Ref | Ref |
| LVEF 41-50% | 804 | 40 | 6.5% (4.8-8.9) | 2.8% (0.6-5.0) | 1.70 (1.16-2.50) | 1.50 (1.02-2.20) |
| LVEF ≤40% | 355 | 58 | 19.5% (15.3-24.7) | 15.8% (11.1-20.5) | 5.96 (4.23-8.41) | 3.61 (2.48-5.26) |
| **Age ≥65** |  |  |  |  |  |  |
| LVEF >50% | 2,564 | 409 | 19.9% (18.2-21.8) | Ref | Ref | Ref |
| LVEF 41-50% | 1,359 | 357 | 32.5% (29.7-35.5) | 12.6% (9.2-16.0) | 1.76 (1.53-2.03) | 1.60 (1.38-1.84) |
| LVEF ≤40% | 1,189 | 465 | 45.8% (42.7-49.1) | 25.9% (22.2-29.6) | 2.88 (2.52-3.29) | 2.36 (2.06-2.71) |

Values are listed as numbers or percentages/ratios with 95% CI.

**Caption:** Table S12 depicts mortality in the three groups of LVEF, grouped by sex and age.

*Adjusted for hypertension, previous ischemic stroke, coronary artery disease, peripheral artery disease, atrial fibrillation, smoking, and diabetes. Additionally, male and female sex were adjusted for age, while age was adjusted for sex.

**Abbreviations:** CI, confidence interval; CIP, cumulative incidence proportion; HR, hazard ratio; LVEF, left ventricular ejection fraction; NSTEMI, non-ST-segment elevation myocardial infarction.
